# Klf4 reduces stemness phenotype, triggers mesenchymal-epithelial transition (MET)-like molecular changes, and prevents tumor progression in nasopharygeal carcinoma

**DOI:** 10.18632/oncotarget.21370

**Published:** 2017-09-27

**Authors:** Xiqing Li, Zhunlan Zhao, Xiaoling Zhang, Sheng Yang, Xia Lin, Xinglong Yang, Xiaolin Lin, Junwen Shi, Shengchun Wang, Wentao Zhao, Jing Li, Fei Gao, Mingyue Liu, Ning Ma, Weiren Luo, Kaitai Yao, Yan Sun, Shengjun Xiao, Dong Xiao, Junshuang Jia

**Affiliations:** ^1^ Guangdong Provincial Key Laboratory of Cancer Immunotherapy Research and Guangzhou Key Laboratory of Tumor Immunology Research, Cancer Research Institute, Southern Medical University, Guangzhou 510515, China; ^2^ Institute of Comparative Medicine & Laboratory Animal Center, Southern Medical University, Guangzhou 510515, China; ^3^ Department of Oncology, The People’s Hosptial of Zhengzhou University, Zhengzhou 450003, China; ^4^ Department of Physiology, Faculty of Basic Medical Sciences, Guilin Medical University, Guilin 541004, China; ^5^ Department of Pathology, The Second Affiliated Hospital, Guilin Medical University, Guilin 541199, China; ^6^ Zhongshan School of Medicine, Sun Yat-sen University, Guangzhou 510080, China; ^7^ Department of Gastroenterology, The First Affiliated Hospital of Jinan University, Guangzhou 510630, China; ^8^ The Third People's Hospital of Shenzhen, Guangdong Medical University, Shenzhen 518112, China

**Keywords:** Klf4, nasopharyngeal carcinoma (NPC), stemness, epithelial-mesenchymal transition (EMT), cancer invasion and metastasis

## Abstract

The reprogramming factor Krüppel-like factor 4 (Klf4), one of the Yamanaka's reprogramming factors, plays an essential role in reprogramming somatic cells into induced pluripotent stem cells (iPSCs). Klf4 is dysregulated and displays divergent functions in multiple malignancies, but the biological roles of Klf4 in nasopharyngeal carcinoma (NPC) remain unknown. The present study revealed that Klf4 downregulation in a cohort of human NPC biopsies is significantly associated with invasive and metastatic phenotypes of NPC. Our results showed exogenous expression of Klf4 significantly inhibited cell proliferation, decreased stemness, triggered mesenchymal-epithelial transition (MET)-like molecular changes, and suppressed migration and invasion of NPC cells, whereas depletion of endogeneous Klf4 by RNAi reversed the aforementioned biological behaviors and characheristics. Klf4 silencing significantly enhanced the metastatic ability of NPC cells *in vivo*. In addition, CHIP assay confirmed that E-cadherin is a transcriptional target of Klf4 in NPC cells. Additional studies demonstrated that Klf4-induced MET-like cellular marker alterations, and reduced motility and invasion of NPC cells were mediated by E-cadherin. This study revealed the clinical correlation between Klf4 expression and epithelial-mesenchymal transition (EMT) biomarkers (including its target gene E-cadherin) in a cohort of NPC biopsies. Taken together, our findings suggest, for what we believe is the first time, that Klf4 functions as a tumor suppressor in NPC to decrease stemness phenotype, inhibit EMT and prevent tumor progression, suggesting that restoring Klf4 function may provide therapeutic benefits in NPC.

## INTRODUCTION

Krüppel-like factor 4 (Klf4) is a member of the Klf family of transcription factors. As one of the Yamanaka's reprogramming factors, Klf4 plays a crucial role in generating induced pluripotent stem cells (iPSCs) [1]. Klf4 protein consists of three zinc finger motifs, which are essential for Klf4 response elements binding, and the N-terminal function as a transcriptional activation domain, the C-terminal as a transcriptional inactivation domain [2, 3]. Klf4 transcriptionally regulates proliferation, differentiation, apoptosis and somatic cell reprogramming in various physiological and pathological processes [1, 4, 5]. Klf4 plays a divergent role in various cancers, depending on different cell context. Evidences from research in gastric adenocarcinoma [6], cervical carcinoma [7], bladder cancer [8], hepatocellular carcinoma [9] and esophageal squamous cell carcinoma [10] indicated that Klf4 functions as a tumor suppressor, whereas Klf4 exerts pro-tumorigenic effects on breast carcinoma [11, 12], colorectal cancer [13], skin squamous cell carcinoma [14], and head and neck squamous cell carcinoma [15]. Moreover, Klf4 plays a critical role in reprogramming non-tumorigenic human mammary epithelial cells into cancer stem cells by defined reprogramming factors [16]. Although loss of cytoplasmic Klf4 protein was frequently observed in the clinical tissue specimens of nasopharyngeal carcinoma (NPC) [17], the functions of Klf4 in NPC is still unknown.

In the present study, we investigate whether Klf4 is involved in stemness, epithelial-mesenchymal transition (EMT) and tumor progression of NPC, as well as the underlying mechanisms. Our findings demonstrate, for the first time, that Klf4, as a tumor suppressor, reduces stemness phenotype, inhibits EMT, and prevents tumor progression in NPC.

## RESULTS

### The reduced Klf4 expression was frequently detected in NPC cell lines and NPC biopsies

**Figure 1:**
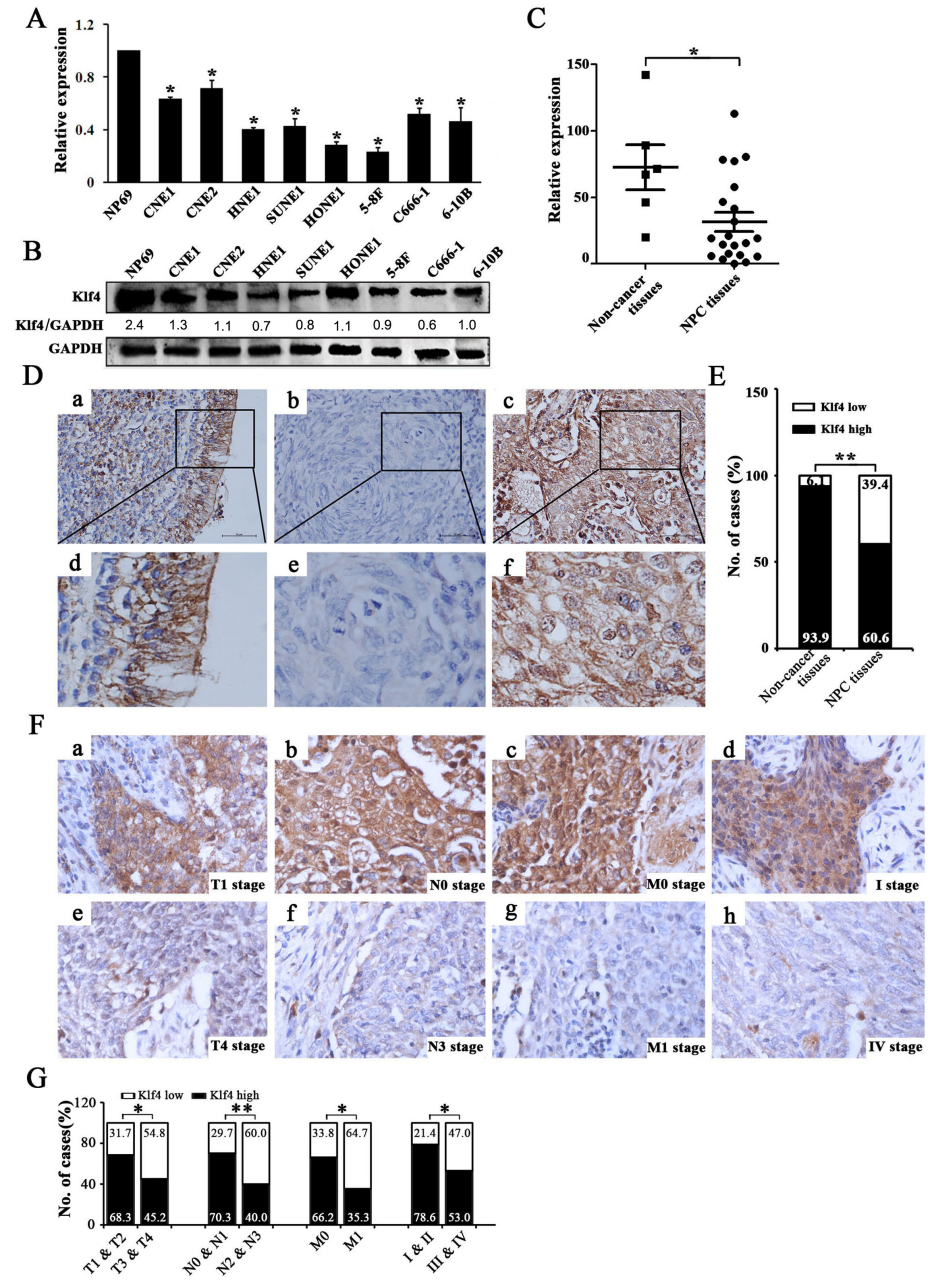
Klf4 was downregulated in NPC cell lines and clinical specimens and Klf4 downregulation was associated with advanced TNM stage of NPC **(A-B)** Expression levels of Klf4 in the indicated 8 NPC cell lines were examined by qRT-PCR (A) and Western blot (B). **(C)** The transcript levels of Klf4 were measured in 21 NPC specimens and 5 noncancerous nasophaygeal epithelial tissues by qRT-PCR. **(D)** Klf4 expression in NPC and non-cancerous nasopharyngeal biopsies based on IHC. a and d: High expression of Klf4 in non-cancerous nasopharyngeal biopsies; b and e: Low expression of Klf4 in NPC biopsies; c and f: High expression of Klf4 in NPC biopsies. The brown staining indicates Klf4 immunoreactivity. **(E)** Klf4 expression was significantly lower in the NPC biopsies than that in the non-cancerous nasopharyngeal biopsies. **(F)** Representative images of Klf4 expression in NPC biopsies of different TNM stages. High expression of Klf4 was observed in the T1 (a), N0 (b), M0 (c) and I (d) stages of NPC biopsies, while low expression of Klf4 was detected in the T4 (e), N3 (f), M1 (g) and IV (h) stages of tumor. **(G)** The numbers and percentages of cases with high or low expression of Klf4 according to different clinicopathological features.

Firstly, we quantitatively evaluated Klf4 expression in NPC cell lines and NPC biopsies using qRT-PCR or Western blotting. Our results showed that the expression of Klf4 was significantly down-regulated in NPC cell lines (Figure 1A, 1B) and NPC biopsies (Figure 1C), compared with controls. Next, we evaluated the expression of Klf4 protein in 94 paraffin-embedded, archived NPC biopsies and 33 paraffin-embedded, archived non-cancerous nasopharyngeal epithelial biopsies using immunohistochemistry (IHC) staining. The results from IHC staining revealed the significantly decreased expression of Klf4 in NPC specimens (Figure 1D, 1E; Supplementary Table 1). Therefore, Klf4 downregulation was more frequently occurred in NPC tissues and cell lines than their non-cancerous counterparts.

### The reduced Klf4 expression was associated with advanced TNM stage of NPC

The relationship between Klf4 expression and a number of clinicopathologic characteristics of NPC patients was summarized in Table 1. No significant association was identified between Klf4 expression and the age (*P*=0.142), sex (*P*=0.665), and histological subtype (*P*=0.207) of 94 NPC cases (Table 1). While Klf4 expression was associated with the tumor size (T classification) (*P*=0.043), lymph node invasion (N classification) (*P*=0.007), distant metastasis (M classification) (*P*=0.027), and clinical stage (*P*=0.023) of 94 NPC patients (Table 1). Briefly, low expression of Klf4 was more frequently observed in T3-T4, N2-N3, M1, and III-IV tumors than T1-T2, N0-N1, M0, and I-II tumors (Figure 1F, 1G and Table 1), indicating that Klf4 loss is a key molecular event in advanced cases of NPC. Taken together, Klf4 may be involved in the progression of NPC.

**Table 1:** Correlation between the clinicopathological features and Klf4 expression in 94 NPC patients

Characteristics	Case No.(n)	Klf4 expression		*χ2*	*P*
		High(*n*,%)	Low(*n*,%)		
Sex					
Female	35	20(57.1)	15(42.9)	0. 285	0.665
Male	59	37(62.7)	22(37.3)		
Age (years)					
<50	44	23(52.3)	21(47.7)	2. 425	0.142
≥50	50	34(68.0)	16(32.0)		
Histological type					
DNKC	6	2(33.3)	4(66.7)	2. 002	0.207
UDC	88	55(62.5)	33(37.5)		
T classification					
T1-T2	63	43(68.3)	20(31.7)	4. 642	0.043
T3-T4	31	14(45.2)	17(54.8)		
N classification					
N0-N1	64	45(70.3)	19(29.7)	7. 863	0.007
N2-N3	30	12(40.0)	18(60.0)		
M classification					
M0	77	51(66.2)	26(33.8)	5. 585	0.027
M1	17	6(35.3)	11(64.7)		
Clinical stage					
I-II	28	22(78.6)	6(21.4)	5. 373	0.023
III-IV	66	35(53.0)	31(47.0)		

DNKC: differentiated nonkeratinizing carcinoma; UDC: undifferentiated carcinoma; T: tumor size; N: lymph node metastasis; M: distant metastasis.

### Klf4 negatively modulated the proliferation of NPC cells

**Figure 2:**
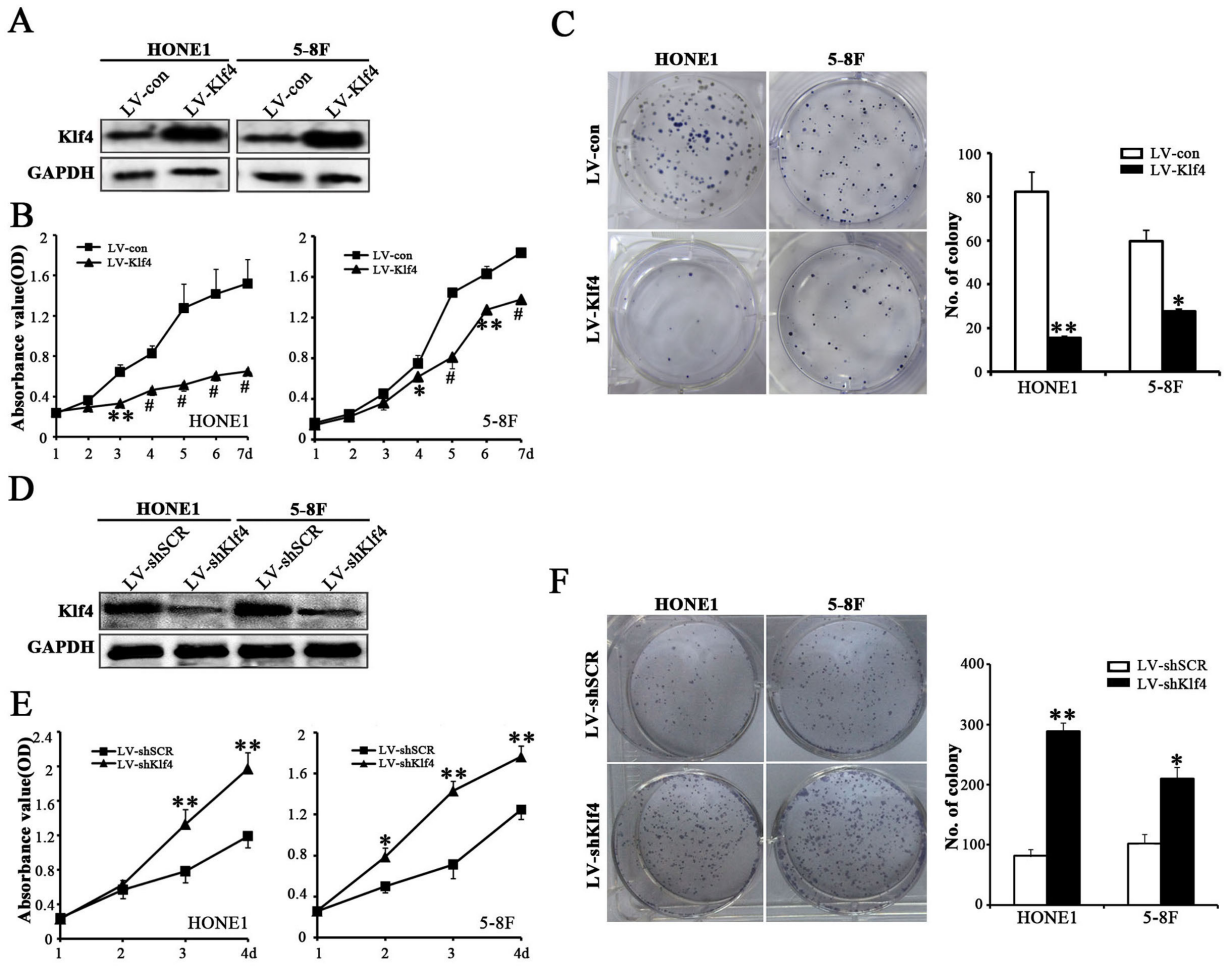
Klf4 negatively regulated the proliferation of NPC cells **(A)** Western blot analysis of Klf4 expression in vector-expressing (LV-con) and Klf4-expressing (LV-Klf4) HONE1 and 5-8F cells. **(B)** The effects of ectopic Klf4 expression on cell proliferation were measured in vector- and Klf4-expressing HONE1 and 5-8F cells by CCK8 assay. **(C)** Colony formation assay was performed to test the proliferation ability of vector- and Klf4-expressing HONE1 and 5-8F cells. The left panels show representative pictures of colony formation assay and the right panels signify the overall counts of the colonies. **(D)** Western blot analysis of Klf4 expression in shSCR-expressing (LV-shSCR) and shKlf4-expressing (LV-shKlf4) HONE1 and 5-8F cells. **(E)** The CCK8 assay was used to evaluate the proliferation of the shSCR- and shKlf4-expressing HONE1 and 5-8F cells. **(F)** Colony formation assay was performed to test the proliferation ability of the shSCR- and shKlf4-expressing HONE1 and 5-8F cells.

Given that the data from T classification (Figure 1F, 1G and Table 1) illustrated that Klf4 is significantly downregulated in larger sized tumors, we suspected that Klf4 may play an essential role in tumor growth, which prompted us to perform gain-of-function and loss-of-function experiments to further explore the effects of Klf4 on NPC cell growth by CCK8 assay and colony formation assay. The Klf4 transgene was successfully over-expressed in HONE1 and 5-8F cells (Figure 2A), while the shRNA-Klf4 specifically knocked down endogenous Klf4 protein expression in both HONE1 and 5-8F cells (Figure 2D). CCK8 assay (Figure 2B), colony formation assay (Figure 2C) and cell cycle analysis (Supplementary Figure 1) revealed that Klf4 overexpression resulted in significantly decreased NPC cell growth. Conversely, CCK8 (Figure 2E) and colony formation (Figure 2F) assays also demonstrated that knockdown of endogenous Klf4 by RNA interference (RNAi) markedly enhanced the proliferation of HONE1 and 5-8F cells. Summarily, Klf4 negatively regulates NPC cell proliferation *in vitro*.

### Klf4 negatively regulated the stemness of NPC cells

**Figure 3:**
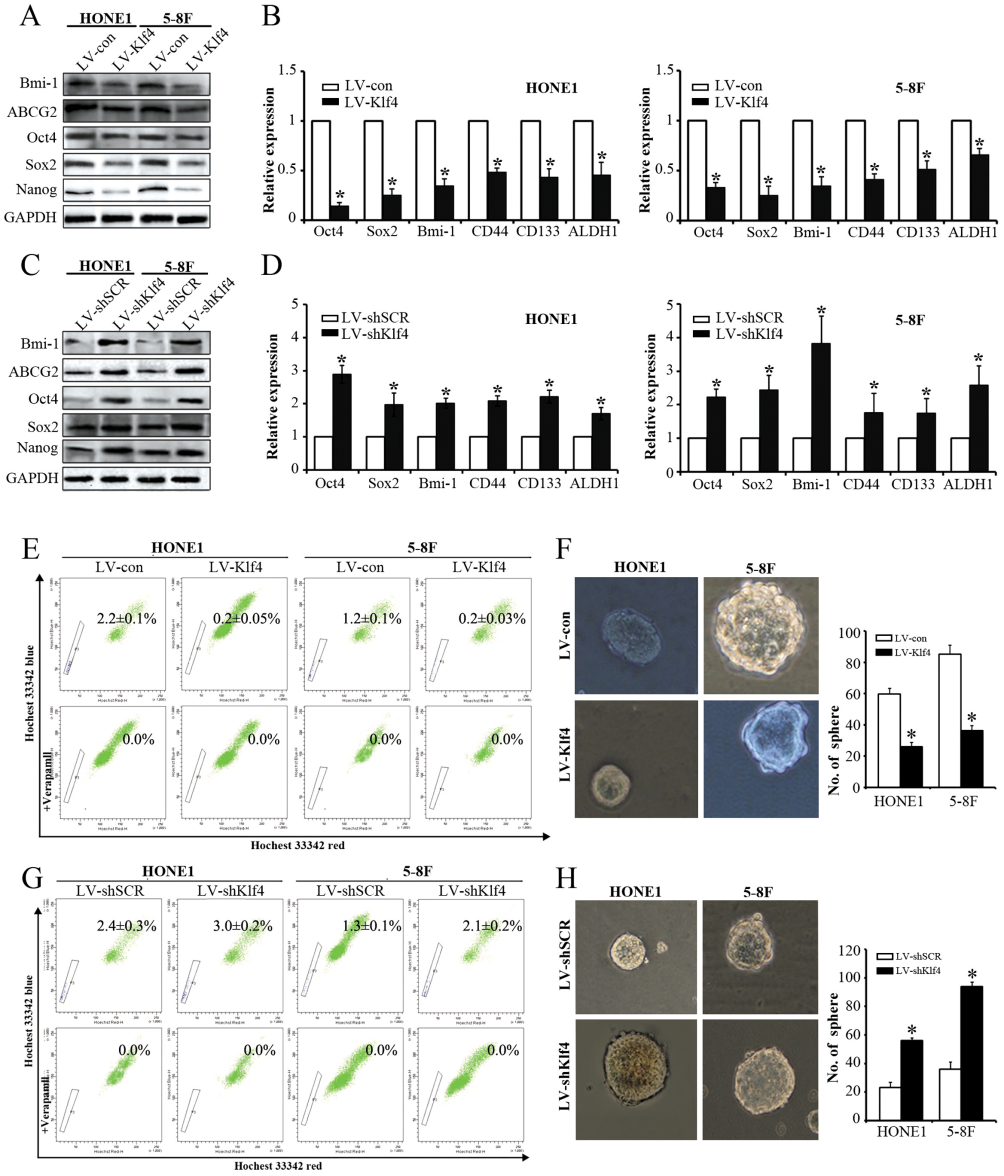
Klf4 negatively modulated the stemness of NPC cells **(A-B)** Western blot (A) and qRT-PCR (B) analysis of the indicated genes in vector- and Klf4-expressing HONE1 and 5-8F cells. **(C-D)** Expression of the indicated genes in shSCR- and shKlf4-expressing HONE1 and 5-8F cells was tested by Western blot (C) and qRT-PCR (D). **(E)** Flow cytometry analysis of the percentages of SP cells in vector- and Klf4-expressing HONE1 and 5-8F cells. **(F)** Flow cytometry analysis of the percentages of SP cells in shSCR- and shKlf4-expressing HONE1 and 5-8F cells. **(G)** Klf4 overexpression in HONE1 and 5-8F cells suppressed tumor spheroid formation. **(H)** Depletion of endogeneous Klf4 in HONE1 and 5-8F cells promoted tumor sphere formation.

Since our results showed that Klf4 negatively modulates NPC cell proliferation *in vitro*, we further determine the effects of Klf4 on stem cell-like populations in NPC by detecting stemness markers, SP cells (side population cells) detecting assay and tumorsphere formation assay. Ectopic expression of Klf4 in NPC cells led to the downregulation of stem cell-related markers (i.e., Bmi-1, ABCG2, Oct4, Sox2, Nanog, CD44, CD133 and ALDH1) at mRNA and/or protein levels (Figure 3A, 3B), whereas depletion of endogeneous Klf4 by RNAi caused the upregulation in the aforementioned stemness markers (Figure 3C, 3D). Additionally, SP cells among NPC cells and tumorspheres have been reported to exhibit CSC characteristics [18]. We first tested the effects of Klf4 overexpression on the percentages of SP cells in HONE1 and 5-8F cells, and found that exogenous expression of Klf4 dramatically decreased the percentage of SP cells in HONE1 cells (0.2% vs. 2.2%, compared with the control) and 5-8F cells (0.2% vs. 1.2%, compared with the control) (Figure 3E). On the contrary, RNAi-mediated silencing of endogenous Klf4 significantly enhanced the percentage of SP cells in HONE1 (3.0% vs. 2.4%, compared with the control) and 5-8F cells (2.1% vs. 1.3%, compared with the control) (Figure 3G). Subsequently, we further examined the ability of NPC cells to form tumor spheres after Klf4 overexpression or Klf4 silencing by RNAi. Sphere-forming assays indicated that the ectopic Klf4 expression led to the decrease in sphere number (Figure 3F). Conversely, silencing of endogenous Klf4 caused the increase in sphere number (Figure 3H). Thus, these findings demonstrate that Klf4 negatively modulates the stemness of NPC cells.

### Klf4 suppressed tumor growth of NPC cells in nude mice

**Figure 4:**
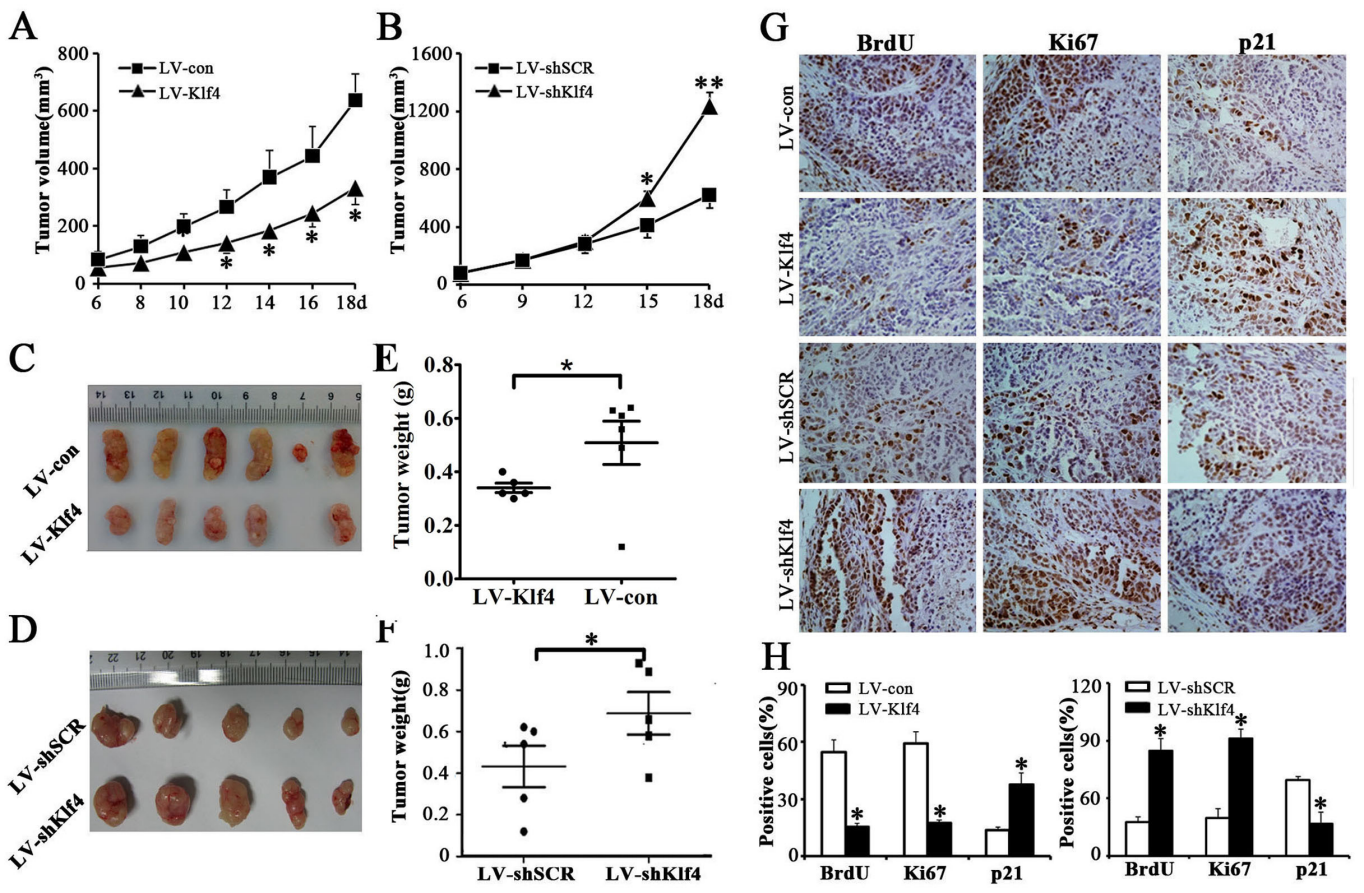
Klf4 suppressed tumor growth of NPC cells in nude mice As mentioned in Materials and methods section, nude mice were subcutaneously implanted with vector- and Klf4-expressing HONE1 cells (A,C,E,G,H) or shSCR- and shKlf4-expressing HONE1 cells (B,D,F,G,H). **(A-B)** Growth curve of tumor volumes. **(C-D)** Representative picture of tumors formed. **(E-F)** Tumors were weighted. **(G-H)** BrdU, Ki67 and p21-stained sections of transplanted tumors formed by HONE1 cells. The percentages of BrdU-, Ki67- or p21-positive cancer cells were calculated by the total number of BrdU-, Ki67- or p21-positive cells over total number of cancer cells.

To further confirm the growth-inhibiting effects of Klf4 on NPC cells *in vivo*, we constructed xenograft models in nude mice. Vector- and Klf4-expressing HONE1 cells or shSCR- and shKlf4-expressing HONE1 cells were injected subcutaneously into the dorsal flank of nude mice. The tumors became palpable 6 days after inoculation (Figure 4A, 4B). The tumor volume (Figure 4A), tumor size (Figure 4C) and tumor weight (Figure 4E) were significantly larger in tumors induced by vector-expressing cells compared with tumors induced by Klf4-expressing cells. Conversely, depletion of endogeneous Klf4 markedly accelerated tumor growth *in vivo* (Figure 4B, 4D, 4F). Additionally, the results of immunohistochemical analysis revealed that the numbers of hyperproliferative BrdU- and Ki67-positive tumor cells in Klf4-expressing cells were significantly decreased, and the number of p21(cyclin-dependent kinase inhibitor)-positive tumor cells in Klf4-expressing cells was significantly increased, compared with control (Figure 4G, 4H). In contrast, depletion of endogeneous Klf4 caused higher proliferation index and downregulation of p21 in xenografts (Figure 4G, 4H). Taken together, these findings demonstrate that Klf4 can suppress *in vivo* tumorigenicity of NPC cells.

### Klf4 inhibited EMT-like cellular marker alterations and reduced the migration and invasion of NPC cells

**Figure 5:**
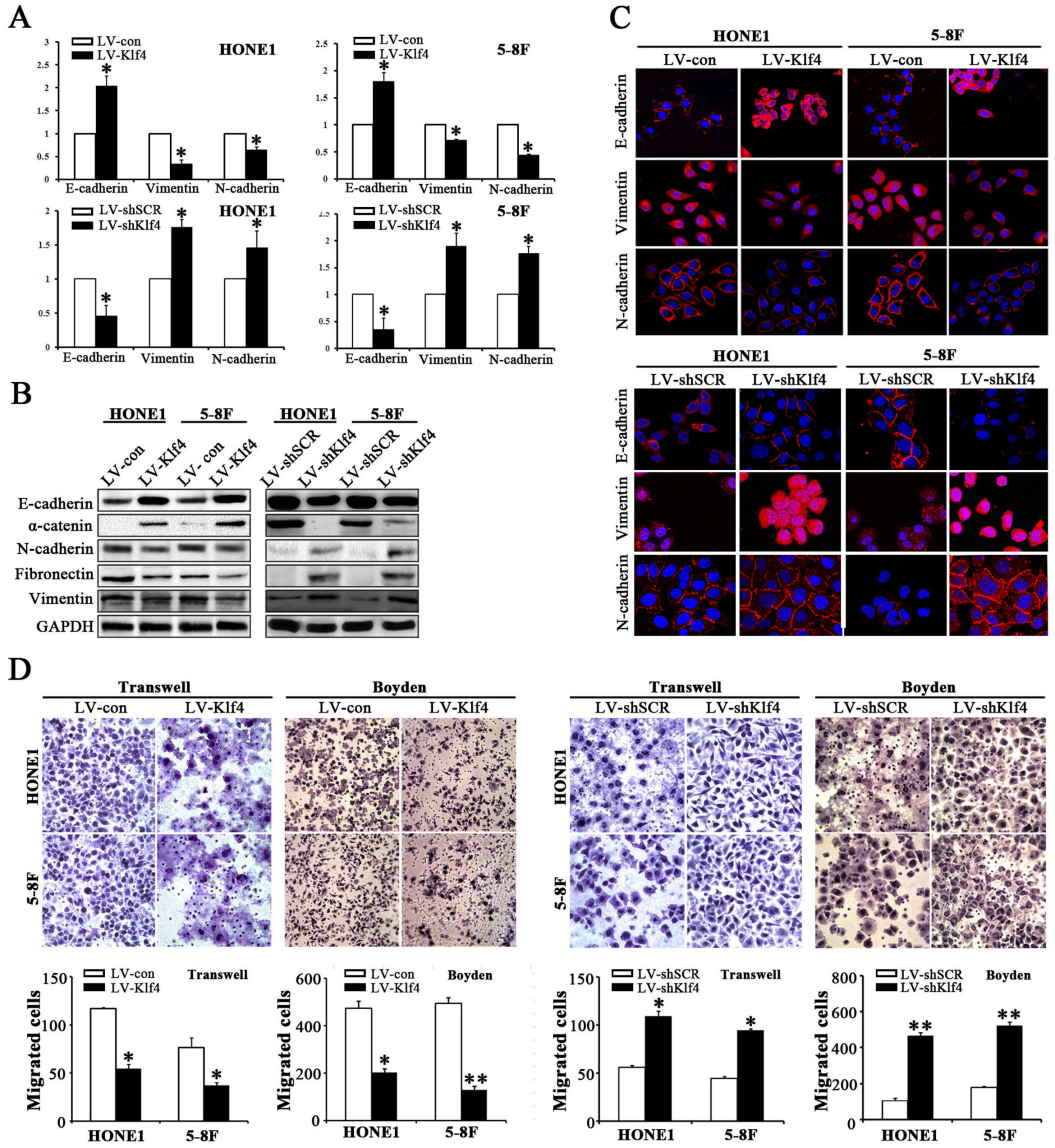
Klf4 inhibited EMT-like molecular changes and reduced the migration and invasion abilities of NPC cells *in vitro* **(A)** The mRNA levels of the indicated genes in Klf4-expressing or shKlf4-expressing NPC cells based on qRT-PCR. **(B)** The protein levels of the indicated genes in Klf4-expressing or shKlf4-expressing NPC cells based on Western blot. **(C)** The expression levels of E-cadherin, vimentin and N-cadherin in Klf4-expressing or shKlf4-expressing NPC cells based on immunofluorescent staining. **(D)** The motile and invasive activities of Klf4-expressing or shKlf4-expressing NPC cells based on transwell migration and Boyden invasion assays, respectively. The average number of cells per field were from 3 repeated independent experiments (Original magnification: ×200).

As shown in Figure 1F, 1G and Table 1, the data from human NPC specimens illustrated that Klf4 downregulation correlates with lymph node involvement and metastasis in NPC. EMT play a central role in invasion and metastasis of various cancers [19]. To illustrate the roles of Klf4 in EMT of NPC cells, we detected the changes of epithelial and mesenchymal markers in NPC cells with ectopic Klf4 expression or endogenous Klf4 silencing by RNAi. qRT-PCR revealed that ectopic expression of Klf4 dramatically increased the mRNA expression of epithelial marker E-cadherin and remarkably reduced the expression of mesenchymal markers (vimentin and N-cadherin) in HONE1 and 5-8F cells (Figure 5A, up). Furthermore, Western blotting results also demonstrated that Klf4-expressing cells displayed typical epithelioid phenotypes [i.e., mesenchymal-epithelial transition (MET)-like molecular changes], including upregulation of epithelial markers (E-cadherin and α-catenin) and downregulation of mesenchymal markers (N-cadherin, fibronectin and vimentin) (Figure 5B, left). Immunofluorescence results also showed the upregulation of E-cadherin and downregulation of N-cadherin and vimentin (Figure 5C, up). Conversely, depletion of endogenous Klf4 resulted in down-regulated epithelial markers and up-regulated mesenchymal markers (Figure 5A, down; Figure 5B, right; Figure 5C, down). Subsequently, we also examined the effects of Klf4 on the motility and invasion of NPC cells by transwell migration assay, Boyden invasion assay and/or wound healing assay. As shown in Figure 5D and Supplementary Figure 2, Klf4-expressing cells displayed significantly reduced mobility and invasion abilities, whereas silencing of endogenous Klf4 in NPC cells induced enhanced mobility and invasion abilities. Meanwhile, exogenous expression of Klf4 significantly enhanced cell adherence in HONE1 and 5-8F cells (Supplementary Figure 3). Taken together, these results suggest that Klf4 suppresses the mobility and invasion of NPC cells *in vitro* by triggering mesenchymal-epithelial transition (MET)-like molecular changes.

### Silencing of endogenous Klf4 promotes the metastasis of NPC cells *in vivo*

**Figure 6:**
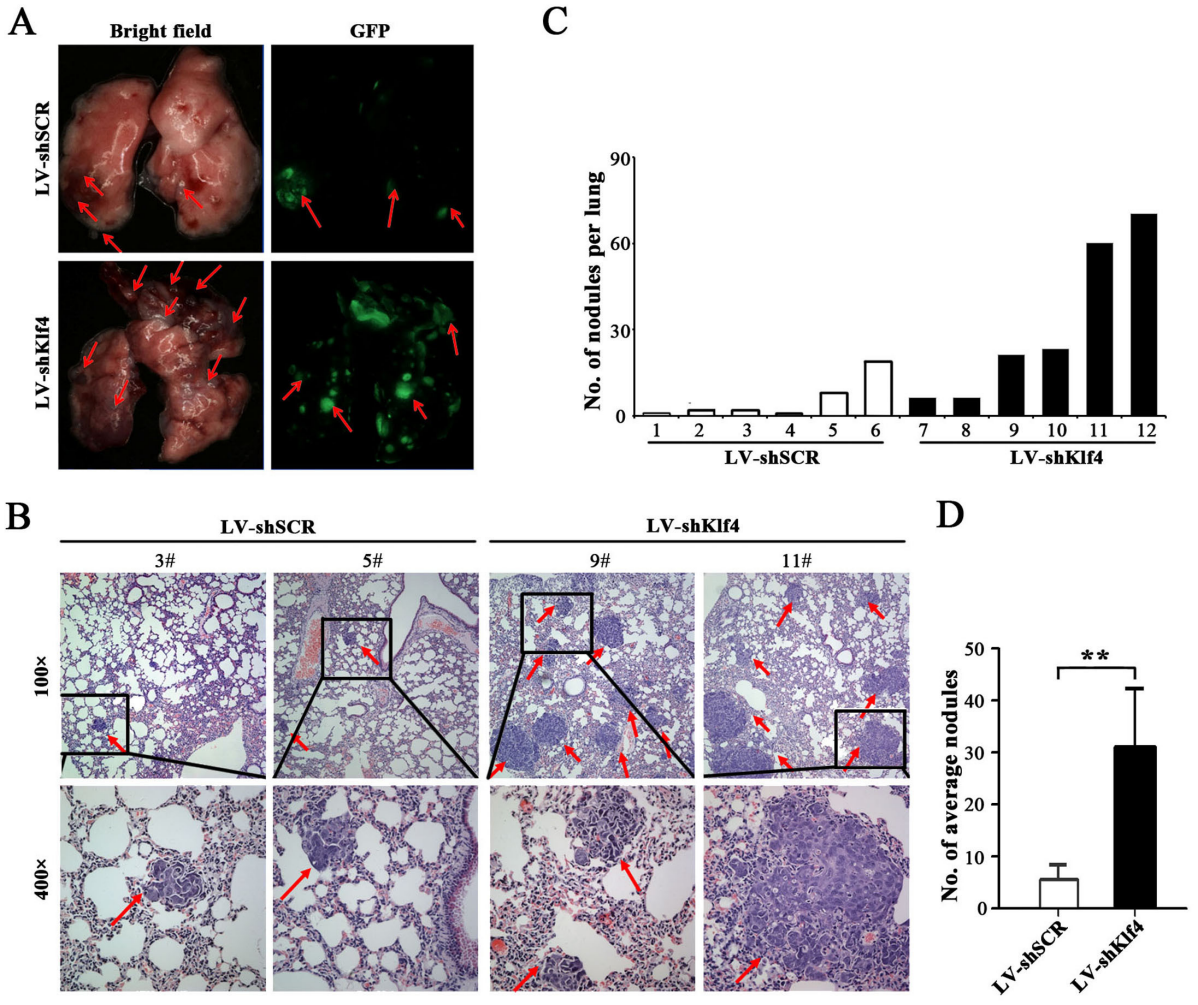
RNAi-mediated silencing of endogenous Klf4 promoted the metastasis of NPC cells *in vivo* **(A)** Representative photographs of lung from nude mice 30 days after subcapsular transplantation of vector- or shKlf4-expressing HONE1 cells into liver. Picture is recorded under natural light (left) or under fluorescent microscopes (right). Red arrows indicate metastatic tumor nodules. **(B)** Representative H&E staining of lung sections of nude mice 30 days after transplantation. Red arrow indicates the metastatic nodules. **(C)** The number of metastatic nodules in lungs of each nude mouse. **(D)** The average number of metastatic nodules was counted. The number of spontaneous lung metastatic nodules in nude mice based on 10 serial sections with 4 μm thick per sample.

Local invasion and distant metastasis are common clinical features of NPC [20]. To determine whether Klf4 is involved in the metastasis of NPC cells *in vivo*, Klf4-depleted HONE1 cells were subcapsularly transplanted into the liver of nude mice and the metastatic tumors on lung surface were evaluated. Compared with control, we found that metastatic nodules on lung surface of mice injected with Klf4-depleted HONE1 cells exhibited the significant increase in the size and number (Figure 6A). Histological examination identified an average of 31.4±12.7 and 6.33±3.31 metastatic tumor nodules on lung surface in mice received Klf4-depleted and control transplants, respectively (Figure 6B, 6D). The number of metastatic nodules in each mice was summaried in Figure 6C. Collectively, RNAi-medicated silencing of endogenous Klf4 significantly increases the metastatic ability of NPC cells *in vivo*.

### Klf4 triggered MET-like molecular changes and decreased migration and invasion of NPC cells through inducing E-cadherin expression

**Figure 7:**
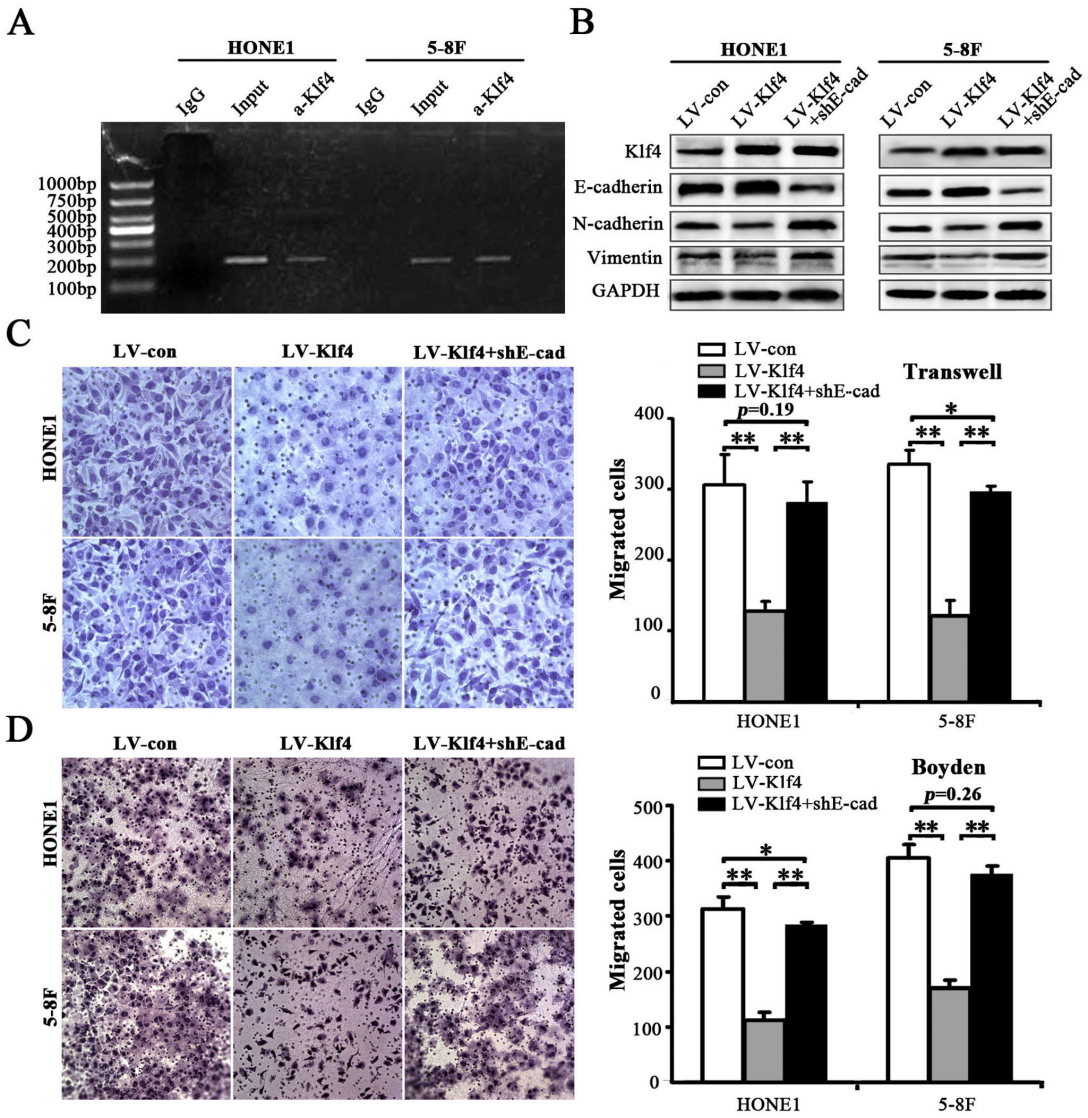
Klf4 suppressed EMT-like cellular marker alterations and decreased migration and invasion of NPC cells through inducing E-cadherin expression **(A)** ChIP assay was performed using anti–Klf4 antibody or IgG antibody to identify Klf4 binding sites on the E-cadherin promoter in HONE1 and 5-8F cells. **(B)** Cell extracts from HONE1 and 5-8F cells infected with LV-con, LV-Klf4 or LV-Klf4 plus shE-cad (E-cad: E-cadherin) were analyzed by immunoblotting with antibodies against the indicated proteins. **(C-D)** Klf4 inhibited the motility (C) and invasion (D) of HONE1 and 5-8F cells through inducing E-cadherin expression. The motile and invasive activities of the indicated NPC cells were analyzed using transwell (C) and Boyden (D) assays, respectively. Original magnification: 200×.

Klf4 was reported to binding to the E-cadherin promoter in human normal and cancerous cells [21, 22]. Loss of cell-cell adhesion, which is mediated by E-cadherin repression, is a landmark of EMT in tumor invasion [23]. In the present study, chromatin immunoprecipitation (ChIP) assay indicated that Klf4 can also bind to the E-cadherin promoter in NPC cells (i.e., HONE1 and 5-8F cells) (Figure 7A). Subsequently, we investigated whether ectopic expression of shE-cadherin (shE-cad) reverses Klf4-induced MET-like molecular changes and rescues Klf4-mediated inhibition of migration and invasion of NPC cells. shRNA-mediated E-cadherin knockdown in Klf4-expressing cells partially or completely rescued the dysruption of EMT-related proteins (including E-cadherin, N-cadherin and vimentin) caused by Klf4 overexpression (Figure 7B). Moreover, transwell migration (Figure 7C) and Boyden invasion (Figure 7D) assays indicated that depletion of endogeneous E-cadherin in Klf4-expressing cells partially or completely abrogated the decreased motility and invasion of HONE1 and 5-8F cells induced by the enforced expression of Klf4. Summarily, these results suggest that Klf4 can induce MET-like molecular changes and inhibit the migration and invasion of NPC cells through upregulating E-cadherin expression.

### Association between Klf4 and EMT phenotype in clinical NPC specimens

**Figure 8:**
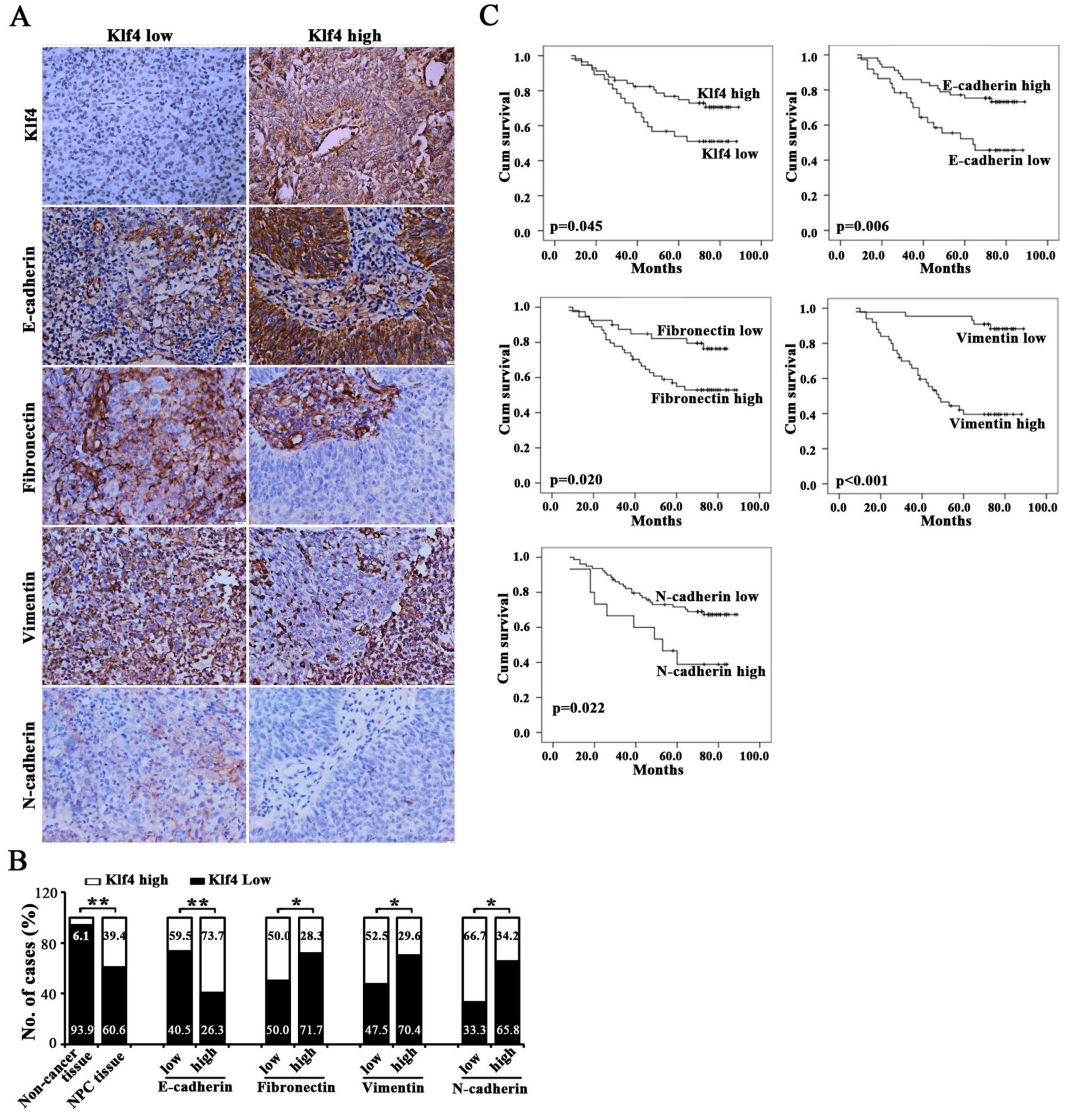
Downregulated Klf4 was associated with EMT phenotype in NPC clinical specimens and poor prognosis **(A)** Representative images reveal that differentially expressed Klf4 was significantly associated with the indicated EMT-related markers in NPC biopsies. **(B)** The numbers and percentages of cases with high or low expression of the indicated genes. **(C)** Cumulative overall survival curves of 94 NPC patients with different expression of indicated proteins (high or low).

We further identified the association between the expression of EMT-related markers (i.e., E-cadherin, Fibronectin, Vimentin and N-cadherin) and Klf4 in 94 cases of human primary NPC tissues. Representative images of immunohistochemical staining in NPC tissues are shown in Figure 8A. Statistical analyses revealed a significant negative association between mesenchymal markers (Fibronectin, Vimentin and N-cadherin) and Klf4 expression, and a significant positive association between E-cadherin and Klf4 expression in NPC biopsies (Figure 8B and Supplementary Table 3). Summarily, Klf4 is also associated with reduced EMT phenotype in human NPC tissues.

Given that Klf4 downregulation is associated with advanced stages of patients, we further evaluated the prognostic value of Klf4 for NPC patients. The patients with lower levels of Klf4 expression had poorer overall survival than those with higher levels of Klf4 expression (Figure 8C and Supplementary Table 2). Besides, the patients with higher levels of E-cadherin expression had better overall survival, whereas the patients with higher levels of mesenchymal marker expression (including Fibronectin, Vimentin and N-cadherin) had poorer overall survival. Moreover, univariate analyses showed that high expression of Klf4 was an independent prognostic factor for OS (Supplementary Table 2). Although multivariate analysis showed that Klf4 was not an independent prognostic factor for OS, the *P* value only narrowly missed the significance (*P*=0.088). Together, Klf4 repression is significantly correlated with EMT phenotype and is prognostic for poor survival in NPC patients.

## DISCUSSION

Previous study reported that Klf4 was significantly downregulated in NPC biopsies and cell lines, and loss of Klf4 expression correlated with advanced T & clinical stage of NPC cases and poor prognosis of NPC [17], which is consistent with the results of this study. To our knowledge, the present study was the first one reporting that Klf4 expression is associated with the invasion and metastasis of human NPC, such as lymph node metastasis (N classification) and distant metastasis (M classification), which has never been reported in NPC. Collectively, Klf4 loss in the advanced stages suggests an essential role of Klf4 in the progression of NPC, however, it’s biological functions and underlining mechanisms in NPC progression still need to be elucidated.

Although Klf4 is noted for its role in somatic cell reprogramming [1], Klf4 was identified initially as a gene of growth arrest [2]. Constitutive expression of Klf4 resulted in the inhibition of DNA synthesis [2] and colony formation [24]. The inducible expression of Klf4 in the inducible RKO cell line led to cell cycle arrest at G1/S checkpoint, accompanied by the activation of expression of p21WAF1/Cip1 [25], a potent suppressor of cell proliferation [26]. Actually, loss of Klf4 has been observed in many types of human tumors, including acute myeloid leukemia [27], lymphoma [28], prostate cancer [29], bladder cancer [8] and neuroblastoma [30], and restored Klf4 expression inhibited cell proliferation in these cancer cells [30]. Klf4 overexpression in neuroblastoma cell SH-SY-5Y upregulated cell-cycle inhibitor protein p21(WAF1/CIP1), and knocking down p21 could partially rescue the suppressive effect of Klf4 [30]. The T classification data from the human NPC biopsies of this study and previous study [17] evidently revealed that Klf4 is significantly downregulated in larger sized tumors, indicating the tumor-suppressive role of Klf4 in NPC, as confirmed by this study. In the present study, gain-of-function and loss-of-function experiments demonstrated that Klf4 negatively modulated the proliferation of NPC cells *in vitro* and *in vivo*. Actually, in xenografted tumors, ectopic expression of Klf4 correlated with smaller tumor size, while Klf4 silencing correlated with larger tumor size.

Klf4 is famous for its reprogramming function in inducing pluripotent stem cells. Klf4 was initially identified by Yamanaka et al. [1] and also confirmed by Jaenisch et al. [31] as a factor for the generation of iPSCs. Klf4 were then proposed to play a essential role in cancer stem cells (CSCs). The previous studies showed that Klf4 is essential for maintaining CSC-like cells in colon CSC-enriched spheroid cells [13] and breast cancer stem cells [32], whereas Klf4-mediated suppression of CD44 signaling negatively modulated the stemness of pancreatic cancer [33].

To date, it is still unknown how Klf4 functions in NPC CSCs. In keratocytes, the normal counterpart of most subtypes of NPC, Klf4 promote the expression of involucrin, a gene that is specifically expressed in differentiated keratinocytes [34]. Meanwhile, ACTL6a enforced the epidermal progenitor state by suppressing SWI/SNF-dependent induction of Klf4 [35]. The above data implies that Klf4 can negatively modulate stemness and positively promote differentiation of keratocytes. Actually, our results from this study, for the first time, revealed that Klf4 negatively regulated the stemness of NPC cells, which is in line with the functions of Klf4 on stemness in its normal counterpart. Since CSCs are tumorigenic (tumor-forming), and hypothesized to cause relapse and metastasis by giving rise to new tumors, the functions of Klf4 in NPC CSCs may also contribute to the tumorigenesis and metastasis of NPC cells in our study.

Our present study strongly supported loss of Klf4 associated with the invasion and metastasis phenotype in 94 NPC cases. Gain-of-function and loss-of-function studies demonstrated that Klf4 negatively modulated the migration and invasion of NPC cells *in vitro*. Furthermore, Klf4 silencing significantly enhanced the metastatic ability of NPC cells in mouse NPC model. These findings strongly support the role of Klf4 in invasion and metastasis of NPC.

Cancer metastasis is a multistep process associated with the induction of an EMT [19, 36]. Data from multiple cancers indicated that Klf4 can have divergent functions in EMT. It was reported that Klf4 acts as a transcriptional activator of epithelial genes and as a repressor of mesenchymal genes to inhibit the EMT process, migration and invasion of breast cancer cells [37]. In addition, the similar results were also found in other maligancies, including non-small-cell lung cancer (NSCLC) [38], hepatocellular carcinoma [39], ovarian cancer [40], renal cell carcinoma [41] and gastrointestinal cancer [42]. In contrast to the above-mentioned findings, Klf4 promotes Twist1-mediated EMT in urothelial carcinoma of bladder [43]. In this study, we found that ectopic expression of Klf4 resulted in MET-like molecular changes and inhibition of migration and invasion in NPC cells, whereas silencing endogenous Klf4 expression reversed MET-like phenotypes and enhanced cell motility and invasion. Moreover, the previous studies revealed that up-regulation of EMT markers (i.e., vimentin, fibronectin, snail and slug), and E- to N-cadherin switch, occurred preferentially in tumors containing a large proportion of spindle-shaped malignant cells [44, 45]. Thus, in the present study, we examined the expression of Klf4 and EMT biomarkers in a larger cohort of 94 NPC samples to understand the clinical correlation between Klf4 expression and EMT biomarkers. Our data illustrated that high expression of vimentin, fibronectin and N-cadherin, and the low expression of E-cadherin were significantly associated with the loss of Klf4 expression in EMT, invasion and metastasis of NPC, indicating that Klf4 plays an important role in triggering MET in NPC specimens, which was consistent with the findings of *in vitro* studies described above. Collectively, we demonstrate that dysrupted Klf4 plays an important role in the pathogenesis of NPC by inducing EMT-like cellular marker alterations to promote the invasion and metastasis of NPC cells.

E-cadherin was identified as a transcriptional target of Klf4 in normal and malignant cells [22, 37, 46, 47]. Our results also showed that Klf4 transactivated E-cadherin by binding the promotor of E-cadherin gene in NPC cells. E-cadherin is a calcium-dependent cell-cell adhesion glycoprotein and organizes the adherent junctions of the epithelia. Loss of E-cadherin dissembles the cadherin-catenin complex of adherent junctions, allowing epithelia cells to migrate and invade [48]. In fact, loss of E-cadherin function or expression has been implicated in cancer progression and metastasis [23]. The downregulation of E-cadherin, which might be due to hypermethylation of E-cadherin promoter or its downregulation by cellular transcription repressor, are observed in NPC [49]. In this study, a new mechanism of E-cadherin loss, which is mediated by down-regulated expression of Klf4 in NPC, is in line with the previous reports in various cancers [22, 37, 46, 47].

Besides E-cadherin, several other EMT-related factors, such as Snail1, Slug, Twist, ZEB1 and vimentin, were also reported as direct or indirect targets of Klf4 [38, 39]. In order to elucidate the roles of E-cadherin in Klf4-mediated MET phenotype and inhibition of the migration and invasion, we carried out the ectopic expression of shE-cadherin in Klf4-expressing NPC cells, and found that ectopic expression of shE-cadherin partially or completely rescued the dysruption of MET-related proteins and abrogated the inhibition of the motility and invasion of NPC cells induced by Klf4 overexpression. Therefore, these data imply that E-cadherin is a key executor of Klf4-mediated MET phenotype and the decreased motility and invasion.

In line with its biological functions revealed by the present study, low expression of Klf4 is a poor prognostic predictor for NPC. Patients with low Klf4 showed poor overall suvival. In addition, univariate analysis exhibited that low Klf4 is associated with a short overall suvival and an independent prognostic predictor. Together, Klf4 loss, which leads to increased stemness phenotype and EMT, contributes to the progression of NPC.

Additionally, the accumulating evidence indicates that epithelial-mesenchymal transition (EMT) can increases the number of cancer stem-like cells in cancer cells [50–52], while the recent study revealed that activation of PKA leads to mesenchymal-epithelial transition (MET) and thereby loss of tumor-initiating ability [53], which provides proof-of-principle for inducing an MET as differentiation therapy for tumor-initiating cells (TICs). In this study, the ectopic expression of Klf4 in NPC cells triggered MET-like molecular changes and decreased stemness features of NPC cells, whereas RNAi-based Klf4 silencing induced EMT-like cellular marker alterations and increased stemness features of NPC cells. The aforementioned information and our findings suggest the causal relationship between Klf4-induced MET and decreased stemness caused by Klf4 overexpression in NPC, which remains to be fully elaborated in the near future.

In conclusion, Klf4 functions as a tumor suppressor in NPC to decrease stemness phenotype, inhibit EMT and prevent tumor progression. Dysrupted Klf4 in NPC can be a diagnostic and prognostic predictor, as well as a therapeutic target of NPC.

## MATERIALS AND METHODS

### Cell lines and cell culture

Human NPC cell lines (including CNE1, CNE2, HNE1, SUNE1, HONE1, 5-8F, 6-10B and C666) and NP69 cells were kindly provided by Prof. Qiao Tao (Chinese University of Hong Kong, Hong Kong, China), Prof. Yixin Zeng (Sun Yat-sen University, Guangzhou, China), and Prof. Musheng Zeng (Sun Yat-sen University, Guangzhou, China). NPC cell lines were cultured in RPMI 1640 medium supplemented with 10% fetal bovine surum (FBS) in a humidified incubator with 5% CO_2_ at 37°C, while NP69 cells was maintained in keratinocyte/serum-free medium (Invitrogen).

### Patients and tissue samples

A total of 94 paraffin-embedded NPC biopsies and 33 non-cancerous nasopharyngeal epithelial biopsies (i.e., chronic nasopharyngitis tissues) (for immunohistochemistry assays), and other 21 fresh NPC specimens and 5 fresh non-cancerous nasophaygeal epithelial tissues (i.e., chronic nasopharyngitis tissues) (for qRT-PCR) were collected in the Department of Pathology, the Second Affiliated Hospital of Guilin Medical College, China, between 2005 and 2009. None of the 94 NPC patients received preoperative radiotherapy or chemotherapy. Informed consent was approved by the local Institutional Research Ethics Committee. The clinicopathologic variables and related information of NPC biopsies were collected as our previously described [54].

### RNA isolation and quantitative real-time PCR (qRT-PCR)

Total RNA was extracted from NPC cells using Trizol Reagent (TaKaRa, Dalian, China) according to the manufacturer’s instruction. Then mRNA was reversely transcribed to cDNA using the PrimeScript RT reagent Kit (TaKaRa). To evaluate the mRNA levels of a number of genes, qRT-PCR was performed on a Stratagene Mx3005P qRT-PCR System using SYBR Green qRT-PCR master mix (TaKaRa). GAPDH was used as the internal control. The primers used in qRT-PCR assay were listed in Supplementary Table 5. All samples were normalized to internal controls and fold changes were calculated based on relative quantification (2^-∆∆Ct^).

### Western blot analysis

Protein lysates were separated by sodium dodecyl sulfate polyacrylamide gel electrophoresis (SDS-PAGE), and transferred to a polyvinylidene difluoride (PVDF) membrane. The blots were probed with the primary antibodies against Klf4, E-cadherin, α-catenin, N-cadherin, vimentin, fibronectin, Bmi-1, ABCG2, Oct4, Sox2, Nanog, followed by HRP (horseradish peroxidase)-labeled secondary antibodies. The hybridization signal was detected using enhanced chemiluminescence (ECL). GAPDH was used as a loading control. The antibodies used in this study were shown in Supplementary Table 6.

### Immunohistochemistry (IHC) staining

After deparaffinization and rehydration, the paraffin-embedded sections were subjected to a high pressure cooker for 2 min for antigenic retrieval. The slides were incubated overnight at 4°C with the following primary antibodies: Klf4 (dilution 1:200), E-cadherin (dilution 1:500), N-cadherin (dilution 1:300), Vimentin (dilution 1:300), BrdU (dilution 1:400), Ki-67 (dilution 1:400) and p21 (dilution 1:300) (Supplementary Table 6).

Serial tumor sections and IHC staining were used to evaluate porteins expression. Klf4, E-cadherin, fibronectin, vimentin and N-cadherin immunoactivity were examined using a mouse polyclonal antibody. BrdU, Ki67 and p21 protein were detected with a mouse polyclonal antibody (Supplementary Table 6).

PBS were used as negative controls. The avidin-biotin technique was applied using DAB for visualization and hematoxylin for counterstaining. Negative controls were prepared by omitting the primary antibody. Histological and IHC evaluation were independently performed by two pathologists without knowledge of the clinicopathological outcomes of the patients. Briefly, each slide was examined in its entirety under a light microscope, and an initial score was assigned which represented the estimated proportion of positive tumor cells (0: ≤5%; 1: 5∼25%; 2: 25∼75%; 3: ≥75%). The score 0, 1 were defined as low and 2, 3 as high. Slides with indeterminate evaluation were re-evaluated, and a consensus was reached, as our previously fully described [55].

### Plasmids, lentivirus production, and transduction

The cDNA of human Klf4 gene (Genebank accession No. NM_004235) was inserted downstream of EF-1α promoter and upstream of IRES-EGFP of lentivirual vector to generate the lentiviral expression vector of pLenti-EF1α-Klf4-IRES-EGFP [56]. The pLenti-shKlf4-GFP was purchased from Applied Biological Materials (ABM) Inc (Canada). The plasmid pLKO.1 puro shRNA E-cadherin was obtained from Addgene (Addgene plasmid 18801). The lentiviral packaging plasmids psPAX2 and pMD2.G were kindly provided by Prof. Didier Trono (University of Geneva, Geneva, Switzerland). To generate stable cell lines, recombinant lentiviruses (namely LV-con, LV-Klf4, LV-shSCR, LV-shKlf4, and LV-shE-cadherin) were generated as previously described [57], and subsequently used to infect HONE1 and 5-8F cells.

### CCK8 assay and colony formation assay

CCK8 assay and colony formation assay were previously fully described [18, 58].

### Percentages of side population cells (SP cells) analyzed by flow cytometry

The changes in the percentage of SP cells in NPC cells were analyzed by flow cytometry (BD FACSAria), as our previously fully described [54].

### Tumor spheroid formation assay

Tumor spheroid formation assay was previously fully described [58].

### Immunofluorescent staining (IF)

For immunofluorescent staining, HONE1 or 5-8F cells grown on the surface of cover slides were transfected with LV-con and LV-Klf4 separately, cells were fixed with 4% paraformaldehyde, rehydrated, and incubated with mouse anti-E-cadherin, anti-N-cadherin (Santa Cruz) and anti-Vimentin (Cell Signaling Technology, Danvers, MA) at room temperature for 40 min. Subsequently, cells were incubated with Alexa Fluor 594-conjugated anti-mouse antibody (Molecular Probes; Invitrogen Corp.) for 40 min at room temperature. The nuclei were stained with DAPI. Slides were examined with a fluorescent confocal microscope (Olympus FV1000, Japan), as our previously fully described [55].

### Transwell migration and Boyden invasion assays

For transwell migration assay, 1x10^5^ cells were seeded into the upper chamber (with 8.0 μm pores, BD Biosciences) in serum-free RPMI 1640. Boyden invasion assay was performed using matrigel (BD Biosciences) in the upper chamber. RPMI 1640 with 10% FBS was loaded in the lower compartment as chemo-attractant. After 20 hours, the migrated or invaded cells were fixed with 100% methanol, stained with hematoxylin solution (Sigma), and counted in five randomly selected optical fields.

### *In vivo* tumorigenicity assay

Nude mice (BALB/C nu/nu) were fed with autoclaved water and laboratory rodent chow. A volume of 100 μL of culture medium mixed with Matrigel (BD Biosciences, San Jose, CA, USA) containing 3×10^6^ HONE1 cells were transplanted into the flanks of mice by subcutaneous injection. The animals were monitored daily, and tumor volumes were measured every 3 days using a caliper slide rule. Tumor volume was calculated as follows: V = 1/2(width^2^ × length). This study was carried out in accordance with the Guide for the Care and Use of Laboratory Animals of the Southern Medical University. The protocol was approved by the Committee on the Ethics of Animal Experiments of the Southern Medical University.

### *In vivo* metastasis analysis in nude mice

Female BALB/c nude mice (4-5 weeks) were purchased from the Medical Laboratory Animal Center of Guangdong Province, and maintained in microisolator cages under aseptic conditions. For *in vivo* metastasis assays, the control or shKlf4-expressing HONE1 cells (1.0×10^6^) were subcapsularly transplanted into the liver of nude mice (6 mice/per group), respectively. All animals were sacrificed on the thirtieth day after transplantation and liver and lung were collected for metastasis analysis. Subcapsular transplantion of NPC cells was performed under sodium pentobarbital anesthesia to minimize suffering.

### Chromatin immunoprecipitation (ChIP)

ChIP assay was performed to identify Klf4 binding sites on E-cadherin promoter in HONE1 cells using the Pierce Agarose Thermo ChIP Kit (Thermo) according to the manufacturer’s instruction. Briefly, cross-linking was performed by adding formaldehyde (final concentration 1%) and incubated at room temperature for 10 minutes. Cross-linking reaction was terminated by the addition of glycine solution. Cells were washed with ice-cold PBS containing 0.1 mM PMSF. Cell pellets were collected by centrifugation at 3000 g for 5 minute and resuspended in 1ml of ChIP sonication buffer. DNA was sheared by sonication and the cell debris was pelleted by centrifugation at 9,000 g for 3 minutes. Equal aliquots of chromatin supernatants were subjected to overnight immunoprecipitation with anti-Klf4 antibody (Sigma-Aldrich) or IgG (negative control). The primer sets used for PCR were listed in Supplementary Table 4.

### Statistical analyses

Statistical analyses were performed using the SPSS 13.0 software package. The χ^2^ test was used to analyze the association between clinicopathological characteristics and Klf4 expression. Multivariate survival analyses were performed with the Cox regression model. Overall survival (OS) was measured from the onset of treatment to the date of death or the survival status at the last date of follow-up. OS probabilities were estimated by the Kaplan-Meier method and the significance of differences was assessed by the log-rank test. Two-tailed Student's t test was used for comparisons of two independent groups. The data were presented as mean ± SEM. Values are statistically significant at ^*^P<0.05; ^**^P<0.01 and ^#^*P*<0.001.

## SUPPLEMENTARY MATERIALS FIGURES AND TABLES


