# Supplementary material for: Klf4 reduces stemness phenotype, triggers mesenchymal-epithelial transition (MET)-like molecular changes, and prevents tumor progression in nasopharygeal carcinoma
Source: Oncotarget. 2017 Sep 27;8(55):93924–41. doi: 10.18632/oncotarget.21370 (PMC5706845; doi:10.18632/oncotarget.21370)
Supplement: Supplementary file 1 [file oncotarget-08-93924-s001.pdf]

Klf4 reduces stemness phenotype, triggers mesenchymal-epithelial transition (MET)-like molecular changes, and prevents tumor progression in nasopharygeal carcinoma

SUPPLEMENTARY MATERIALS

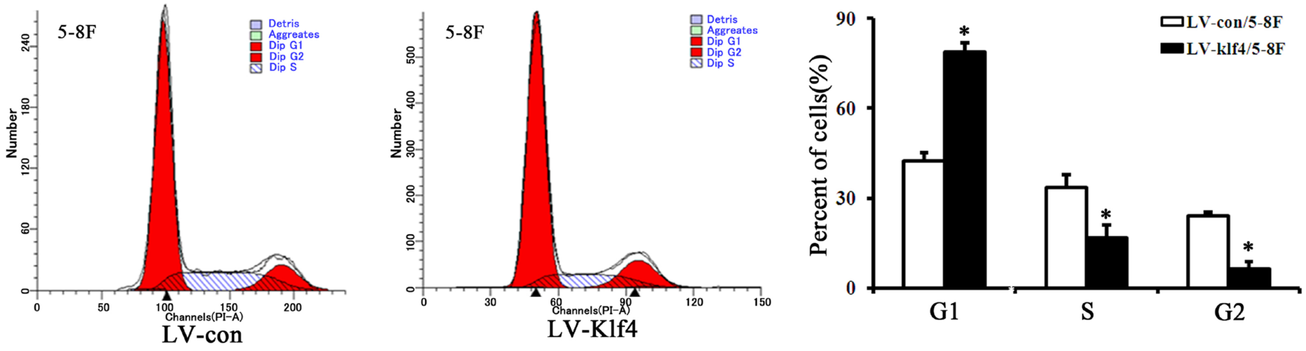

Supplementary Figure 1: Klf4 overexpression increased the G1 phase cells. Klf4 overexpression arrested cell cycle at G1 stage in 5-8F cells. G1 phase cells were 78.5% in klf4-expressing cells (LV-Klf4) and 44.8% in vector-expressing cells (LV-con), respectively.

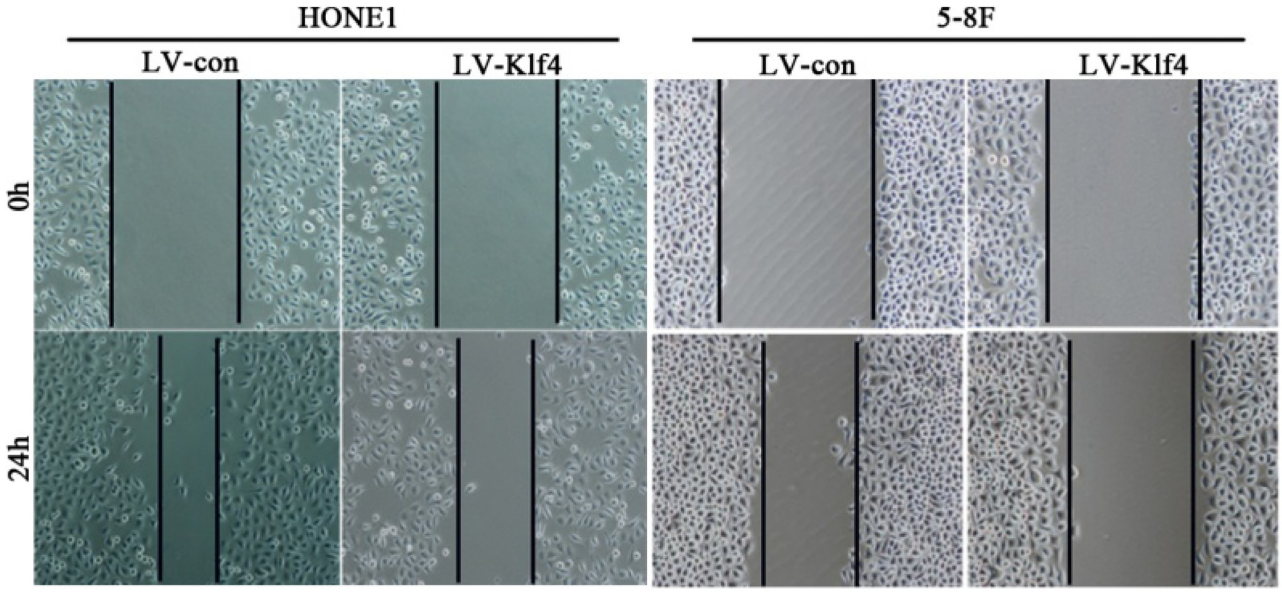

Supplementary Figure 2: Klf4 overexpression inhibited the migration of HONE1 and 5-8F cells. Wound healing assays were performed in HONE1 and 5-8F cells infected with LV-con or LV-Klf4 lentiviruses. Migration activity was determined by measuring the distance from boundary lines of scratch to cell-free space for 48h.

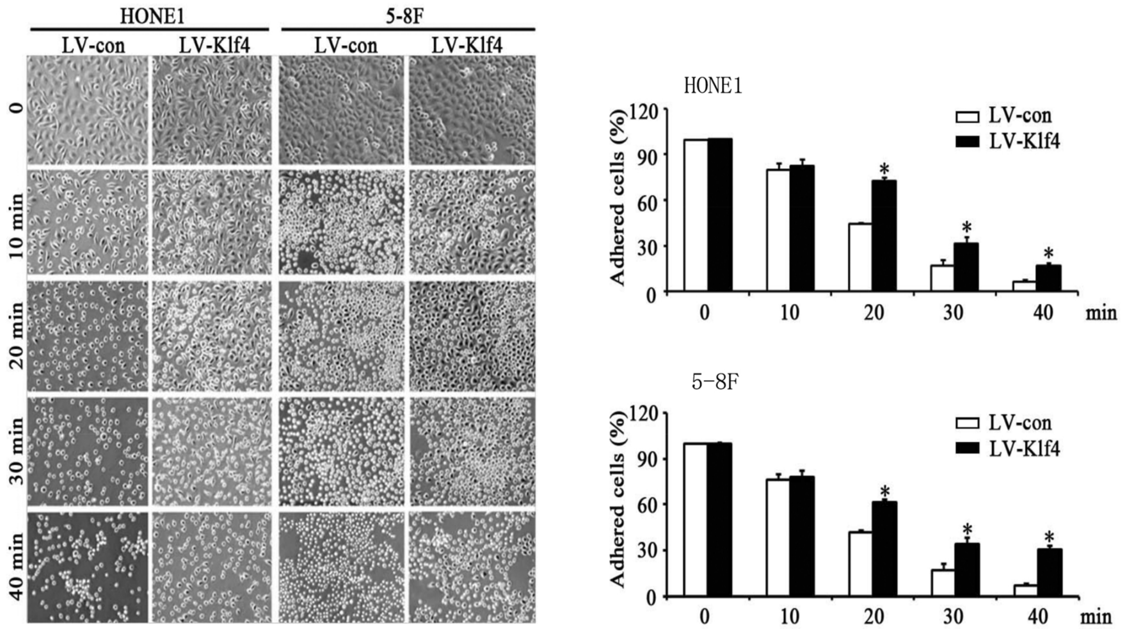

Supplementary Figure 3: Overexpression of Klf4 increased the adhesion of HONE1 and 5-8F cells. Cell adhesion assays were performed in vector- and klf4-expressing NPC cells (i.e., HONE1 and 5-8F cells). Briefly, cells were seeded on culture dishes and grown to ~90% confluence. The confluent monolayer cells were then washed with PBS and treated with 0.05 mM EDTA at 37°C. Cells detachment was examined at different time intervals (10, 20, 30 and 40 min) under a phase contrast microscope. Cell adhesion was analyzed by counting the adherent cells. klf4-expressing cells had higher rates of adhesion than the control cells. *, $P<0.05$ by t test.

Supplementary Table 1: Expression of Klf4 in 33 non-cancerous epithelial tissues and 94 NPC tissues

Variables	*n*	Klf4 expression		χ2	*P*
		Low (*n*, %)	High (*n*, %)		
non-cancerous epithelial tissues	33	2 (6.1)	31 (93.9)	8.762	0.003
NPC	94	37 (39.4)	57 (60.6)		

Supplementary Table 2: Univariate and multivariate analyses of the overall survival of 94 patients with NPC

Variables	Univariate analysis		Multivariate analysis	
	HR (95% CI)	*P*	HR (95% CI)	*P*
Gender				
Female vs. Male	1.615 (0.234-1.953)	0.578		
Age (y)				
<50 vs. ≥50	0.786 (0.378-1.801)	0.654		
Histological type				
DNKC vs. UDC	1.010 (0.426-2.394)	0.982		
T classification				
T1-T2 vs. T3-T4	1.452 (0.654-2.785)	0.547		
N classification				
N0-N1 vs. N2-N3	2.067 (1.130-3.783)	0.033	1.013 (0.455-1.544)	0.877
M classification				
M0 vs. M1	4.578 (2.388-7.965)	0.000	3.054 (1.248-5.987)	0.013
Clinical stage				
I-II vs. III-IV	4.454 (1.578-8.028)	0.021	2.377 (0.878-4.788)	0.387
Klf4 expression				
Low vs. High	2.354(1.325-6.254)	0.008	2.578 (0.784-3.525)	0.088

HR: hazard ratio; 95%CI: 95% confidence interval.

Supplementary Table 3: Association of Klf4 expression and EMT markers in 94 NPC tissues

Variables	*n*	Klf4 expression		*P*
		Low (*n*, %)	High (*n*, %)	
E-cadherin				
Low expression	57	42(73.7)	15(26.3)	0.002
High expression	37	15(40.5)	22(59.5)	
Fibronectin				
Low expression	48	24(50.0)	24(50.0)	0.037
High expression	46	33(71.7)	13(28.3)	
Vimentin				
Low expression	40	19(47.5)	21(52.5)	0.033
High expression	54	38(70.4)	16(29.6)	
N-cadherin				
Low expression	15	5(33.3)	10(66.7)	0.023
High expression	79	52(65.8)	27(34.2)	

Supplementary Table 4: Primers used in ChIP assays

ChIP-primer	Forward primer (5'-3')	Reverse primer (5'-3')
E-cadherin-A	AGGTCTCAGTCCTTTGGCTTGC	TGGTTACACAAGCACCCACATC
E-cadherin-B	GGGAGTGGGAATTTGGAAAG	TCAAAAGGAGGTGGAAGGAT

Supplementary Table 5: Primers used in quantitative real-time PCR (qRT-PCR) assay

Gene	Forward primer (5'-3')	Reverse primer (5'-3')
E-cadherin	TGCCCAGAAAATGAAAAAGG	GTGTATGTGGCAATGCGTTC
Fibronectin	CAGTGGGAGACCTCGAGAAG	TCCCTCGGAACATCAGAAAC
Vimentin	GAGAACTTTGCCGTTGAAGC	GCTTCCTGTAGGTGGCAATC
Sox2	TACAGCATGTCCTACTCGCAG	GAGGAAGAGGTAACCACAGGG
Oct4	CAAAGCAGAAACCCTCGTGC	TCTCACTCGGTTCTCGATACTG
Bmi-1	CCACCTGATGTGTGTGCTTTG	TTCAGTAGTGGTCTGGTCTTGT
CD44	CTGCCGCTTTGCAGGTGTA	CATTGTGGGCAAGGTGCTATT
CD133	AGTCGGAAACTGGCAGATAGC	GGTAGTGTTGTACTGGGCCAAT
ALDH1	CCGTGGCGTACTATGGATGC	GCAGCAGACGATCTCTTTCGAT
GAPDH	ACCCAGAAGACTGTGGATGG	TCTAGACGGCAGGTCAGGTC

Supplementary Table 6: List of antibodies and suppliers used for immunohistochemistry or immunoblotting

Antibody	Isotype	Suppliers
Klf4	Mouse IgG2a	Sigma-Aldrich
E-cadherin	Mouse IgG2a	BD Biosciences
a-catenin	Mouse IgG1	BD Biosciences
Fibronectin	Mouse IgG1	BD Biosciences
Vimentin	Mouse IgG1	BD Biosciences
N-cadherin	Mouse IgG1	BD Biosciences
Bmi-1	Mouse IgG1	Santa Cruz Biotechnology, Inc.
ABCG2	Mouse IgG1	Santa Cruz Biotechnology, Inc.
Oct4	Mouse IgG1	Santa Cruz Biotechnology, Inc.
Sox2	Mouse IgG1	Santa Cruz Biotechnology, Inc.
Nanog	Mouse IgG1	Santa Cruz Biotechnology, Inc.
BrdU	Rabbit Ig	Abgent
Ki67	Rabbit Ig	Abgent
p21	Rabbit Ig	Abgent
GAPDH	Rabbit IgG	Bioss
